# A Case of Calculi with Paralytic Complication

**Published:** 1880-09

**Authors:** Robert W. Westmoreland

**Affiliations:** Atlanta, Ga.


ATLANTA
Medical and Surgical Journal.

Vol. XVIII.] SEPTEMBER—1880. [No. 6.

### Original Communications.

### A CASE OF CALCULI WITH PARALYTIC COMPLICATION.

By ROBERT W. WESTMORELAND, M.D., Atlanta, Ga.

The following case of calculus is deemed sufficiently rare to deserve notice, as showing the extent to which reflex irritability from the presence of the concretion in the bladder may be carried. While it is the usual rule that calculus may exist without manifesting much general nervous irritability in the adult, instances are recorded in which after a period of extended quiescence comparatively, as regards constitutional disturbances, suddenly from some slight cause, considerable results ensue.

Frere Cosme reports the case of a watchmaker whose only symptoms, for a number of years, was an inability to retain his urine. Yet one day in lifting some heavy weight, sudden and violent pain was manifested in the region of the hypogastrium. This continued and became so unbearable that an operation became imperative to relieve his suffering.

Again, Van Helmont relates the case of a priest, who suddenly, following the lifting of a book, experienced considerable pain, and an examination revealed quite a large concretion.

The occasion of these sudden disturbances may be, as a general rule, refered to violent or extensive exertion, or instantaneous shock, which by changing the position of partially encysted calculi, or by producing unusual pressure of the soft parts on those that are not encysted, creates an irritation or inflammation that may lead to constitutional disturbances. Particularly does this tendency bear where the phosphatic calculi exist, for here the irritable and debilitated state of the general system accompanying this character of concretion, renders it more susceptible to reflex influences. In these instances the sacro-lumbar nerves by their darting radiating pains give the first warning of approaching complications.

Two years ago Mr. ——, white, suffering with slight inability to hold his urine, received a fall from a horse, striking on the coxcyx. Considerable pain was felt at the time, but he was not disabled from walking, and being a farmer, he carried on his affairs as usual. For awhile, although there existed much pain, and an increase in the incontinence, he was enabled to attend to his ordinary affairs. Shortly, however, in connection with his other difficulties, symptoms of paralysis began to exhibit themselves. This state of affairs progressed until finally he lost almost entire use of his lower limbs, and was compelled to resort to crutches. In addition to these paralytic difficulties, the spells of incontinence were capricious, it being necessary to use a catheter at times to draw off the urine. A peculiar symptom is that the patient complains of a feeling of fullness, as if there were a large lump just at the lower edge of the liver. He describes it as if there were a pain darting down from this lump to the perineum, occurring in paroxysms. The patient is able to stand with eyes closed, and can hobble along without crutches, but to cross the legs and strike just below the patella, or even any other part of the leg there will occur sudden and extensive contraction of the extensor muscles.

Upon physical examination, there was discovered what was supposed to be a good sized stone, with greatly hypertrophied prostate. The patient was advised to undergo an operation for the removal of stone as soon as circumstances would allow, with the assurance that probably all difficulties would gradually disappear thereafter.

We hope at some future time to be able to report favorable progress in this case, or at least to demonstrate the connection, if any, between the concretion and paralysis.

